# A comparison of statistical methods for deriving occupancy estimates from machine learning outputs

**DOI:** 10.1038/s41598-025-95207-3

**Published:** 2025-04-27

**Authors:** Lydia K. D. Katsis, Tessa A. Rhinehart, Elizabeth Dorgay, Emma E. Sanchez, Jake L. Snaddon, C. Patrick Doncaster, Justin Kitzes

**Affiliations:** 1 School of Geography and Environmental Science, University of Southampton, Southampton, UK; 2 Department of Biological Sciences, University of Pittsburgh, Pittsburgh, PA USA; 3 Ya’axché Conservation Trust, Punta Gorda Town, Toledo District, Belize City, Belize; 4 Panthera-Belize, Panthera Wild Cat Conservation Belize, 5189 Monday Morning Ave., Cayo District, Belmopan City, Belize; 5 School of Biological Sciences, University of Southampton, Southampton, UK

**Keywords:** Acoustic monitoring, Autonomous recording units (ARUs), Biodiversity monitoring, Yucatán black howler monkey, Occupancy modelling, False-positive models, Biodiversity, Ecological modelling

## Abstract

The combination of autonomous recording units (ARUs) and machine learning enables scalable biodiversity monitoring. These data are often analysed using occupancy models, yet methods for integrating machine learning outputs with these models are rarely compared. Using the Yucatán black howler monkey as a case study, we evaluated four approaches for integrating ARU data and machine learning outputs into occupancy models: (i) standard occupancy models with verified data, and false-positive occupancy models using (ii) presence-absence data, (iii) counts of detections, and (iv) continuous classifier scores. We assessed estimator accuracy and the effects of decision threshold, temporal subsampling, and verification strategies. We found that classifier-guided listening with a standard occupancy model provided an accurate estimate with minimal verification effort. The false-positive models yielded similarly accurate estimates under specific conditions, but were sensitive to subjective choices including decision threshold. The inability to determine stable parameter choices a priori, coupled with the increased computational complexity of several models (i.e. the detection-count and continuous-score models), limits the practical application of false-positive models. In the case of a high-performance classifier and a readily detectable species, classifier-guided listening paired with a standard occupancy model provides a practical and efficient approach for accurately estimating occupancy.

## Introduction

Monitoring biodiversity is key to effective conservation management^1^. Identifying spatial and temporal trends of threatened species is particularly important for prioritising conservation action, evaluating the effectiveness of management interventions, and providing feedback for adaptive management^2^. However, species monitoring efforts have long been plagued by imperfect detection, especially failing to detect a species when it is present, which can strongly bias inferences of population trends and responses to environmental covariates^1^.

Many monitoring programmes account for imperfect detection using occupancy modelling^3^. Occupancy modelling relies on repeat observations at each sampling site to estimate the probability of detection and subsequently occupancy, the proportion of sample units estimated to be occupied by the target species^4^. Occupancy analyses are commonly used to infer species’ spatial distributions^5^, population dynamics^1^, and responses to factors such as anthropogenic threats^6^. These inferences have become fundamental for guiding conservation measures for threatened species, including tracking trends in populations over time^7,8^ and identifying priority conservation areas^3^.

Advances in monitoring technology such as camera traps and more recently autonomous recording units (ARUs) enable efficient monitoring of biodiversity at scale^9^. Data collected by ARUs are ideal for occupancy modelling due to the ability to leave sensors unattended, providing replicate surveys at each location. Traditionally, bioacoustic data have been manually reviewed for species presence, and there are well established methods for integrating these data into occupancy models^10,11^. However, machine learning models are increasingly used to process the vast amounts of data collected^12,13^, which assign a score to each sound clip reflecting the model’s confidence in the presence of a target species. While these methods have vastly increased the efficiency of processing acoustic data, there is a lack of clear guidance on integrating the outputs of these classifiers into occupancy models^12,13^.

The challenge in incorporating machine learning outputs into occupancy models largely stems from misidentification of auditory signals by the classifiers^14^. Common practice in machine learning involves converting the continuous score produced by a classifier into a binary class (e.g., present or absent), using a chosen decision threshold. This process typically results in numerous false-positive detections^14,15^ (i.e. an ‘absent’ file being wrongly classified as ‘present’), violating a key assumption of traditional occupancy models^3^. False-positive detections accumulate over time, and even at low rates can lead to severe bias in estimates of occupancy probability^16,17^. Several approaches have been proposed and used for dealing with these types of data. An increasingly recommended approach involves manually verifying species presence in all files above a chosen decision threshold, producing a dataset suitable for traditional occupancy modelling^10,18,19^. However, this method often requires substantial manual effort for data verification and involves selection of a threshold, a subjective choice guided by the user’s priorities to strike a balance between precision (reducing false positives) and recall (reducing missed detections)^20^. More recently, a suite of false-positive occupancy modelling approaches have been developed^21,22^, including some that circumvent the need for a decision threshold, and which promise substantially reduced manual input for removal of false positives^13,23,24^. These approaches likely vary in accuracy, efficiency, and computational requirements, however, there have been no attempts to directly compare these methods by applying them to a single common dataset. Such comparisons are essential for understanding the strengths and weaknesses of available approaches, and for contributing to a wider evidence base that practitioners can draw on for guidance.

In this study we assess the performance of a suite of proposed methods for integrating ARU data and machine learning outputs with occupancy models, using a case study of the Yucatán black howler monkey (*Alouatta pigra*)*,* a vocal primate species endemic to the forests of Central America^25^. We compare alternative occupancy modelling approaches for magnitude and direction of error and further evaluate the efficiency and ease of implementation of each model. We compare: (i) traditional occupancy models with manually verified acoustic data, (ii) binary false-positive occupancy models using presence-absence data^17,26^, (iii) a false-positive occupancy model using counts of detections^23^, and (iv) continuous-score false-positive occupancy models using raw classifier scores^23,24^. We additionally explore the influence of decision threshold, temporal subsampling, and data verification strategies on model estimates. With this case study, we aim to contribute to an important wider evidence base on model performance, which is required to establish more generalisable recommendations for end users.

## Materials and methods

### Study area

**Fig. 1:**
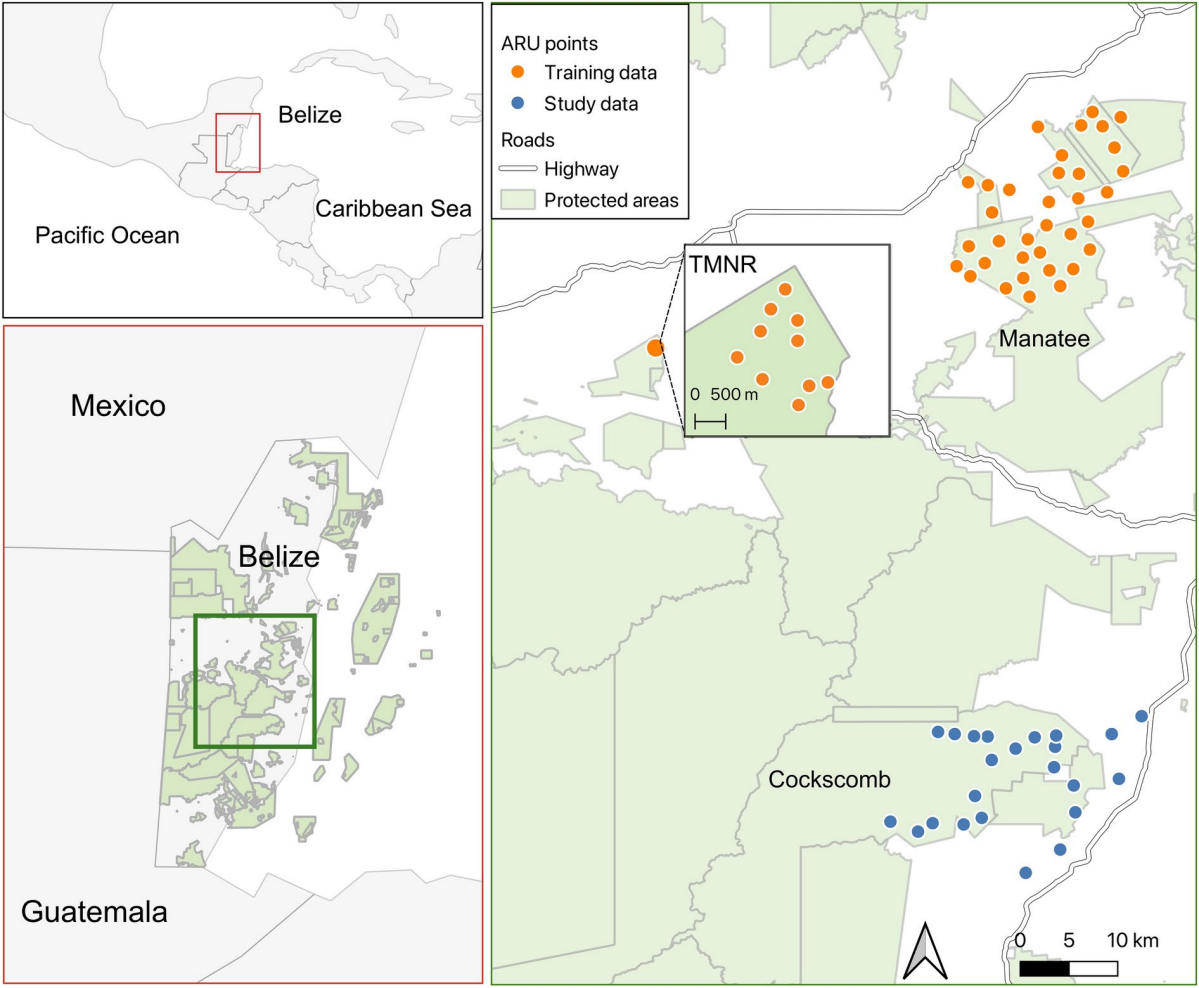
Location of autonomous recorder unit (ARU) deployments in Belize. Data collected in Tapir Mountain Nature Reserve (TMNR; depicted in an inset zoom panel) and Manatee were used for classifier training. Data collected in Manatee and Cockscomb Basin Wildlife Sanctuary (Cockscomb) were used for the occupancy modelling. Map created using QGIS 3.10 (QGIS.org, 2024. QGIS Geographic Information System. QGIS Association. http://www.qgis.org).

Our study areas were situated in central and southern Belize (Fig. 1). The northernmost study area, hereafter referred to as Manatee, comprised Manatee Forest Reserve, several smaller neighbouring protected areas, and several sites outside the protected areas (Fig. 1). The more southerly study area, hereafter referred to as Cockscomb, comprised Cockscomb Basin Wildlife Sanctuary, Mango Creek Forest Reserve and several sites outside the protected areas (Fig. 1). Generally, both areas are characterised by similar habitat types, dominated by broad-leaved moist forest on steep hills, with some lowland savannah^27^. However, Cockscomb has a much steeper elevational gradient, with a peak of 1041 m compared to 593 m in Manatee. Cockscomb also has greater overall forest cover compared to Manatee, which has experienced some forest loss due to hurricane damage and human activity. The final area, Tapir Mountain Nature Reserve (TMNR), was used only to collect training data for the machine learning classifier and was not used to model occupancy. This area is also dominated by broad-leaved moist forest on steep hills.

### Study species

The Yucatán black howler monkey (*Alouatta pigra*) is a primate species native to Belize, eastern Guatemala, and south-eastern Mexico. *A. pigra* is an arboreal, forest-dependent species that lives in groups ranging from 2 to 12 individuals^28^. Home range sizes for this species vary widely, with research in Belize documenting home range sizes between 0.5 and 50 ha^29–31^. Howler monkeys emit loud, low-frequency calls, known as howls, which can be heard over long distances through the forest (> 1 km)^32–34^. Throughout the day, howler monkeys commonly engage in several bouts of howling, with each bout lasting on average around 10–15 min, and sometimes ranging up to an hour^33,35^. The rate of howling bouts is highest around dawn, followed by a smaller peak in the afternoon^33,35^.

### Data collection

We deployed ARUs (AudioMoths^36^) at 10 points in TMNR from March 2018 to March 2019, and at 59 points across Manatee (n = 35) and Cockscomb (n = 24) between January 2021 and June 2021. ARUs were deployed along logging roads and trails, with a minimum separation distance of 2 km, except for in TMNR where device placement was determined by a probabilistic algorithm^37^. ARUs recorded simultaneously within each area, with no rotation of points.

ARUs were all deployed in pairs using the same configuration and setup. At each point, one ARU recorded during the day (07:00–17:00), and the other during the night (17:00–07:00). Devices were deployed primarily to monitor hunting, using custom firmware designed to detect gunshots^38^. The onboard classifier triggered a 4-s recording whenever it detected a gunshot-like sound, characterised by a sharp, broadband blast followed by rapid decay^38^. Howler monkey vocalizations share similar traits, including a sharp onset and broadband signal followed by attenuation^34^. To maximize gunshot detections, the classifier was configured for high recall, resulting in low precision and a high rate of false positives. Consequently, howler monkey vocalizations frequently triggered the detection algorithm and appeared as false-positive gunshot detections in the recordings.

To avoid excessive triggering, a threshold of 100 recordings per hour was set, after which the device stopped recording for the rest of the hour. This threshold was reached roughly 25% of the time at each sensor and there was an average of 69 min of recordings per sensor per day. While data collection was not optimised for howler-monkey detection, ‘messy’ datasets such as these are a reality in conservation^39,40^ and those from surveys using monitoring technology increasingly contain information on by-catch of potential value to conservation^41^.

### Howler monkey classifier

We trained a classifier to provide scores in the range [0, 1] for howler monkey presence within each sound file. We used a convolutional neural network (CNN) for our classification model, as these models have proven to be powerful for sound classification for a broad range of species including howler monkeys^10,19,42^. We curated training data for the model by randomly selecting 1,000 4-s recordings from each of TMNR and Manatee, and reviewing files for howler monkey presence by visually inspecting spectrograms and listening to the audio recordings. We supplemented these data with 7 recordings obtained from the Macaulay Library at the Cornell Lab of Ornithology, which were split into 40 4-s clips containing howler monkey vocalisations following manual review (see acknowledgements for file accreditations). We used this dataset to train a ResNet18 convolutional neural network (CNN) classifier on spectrograms of the 4-s clips. We trained the classifier using OpenSoundscape v0.7.1^43^ (Supplementary Information). We subsequently applied the classifier to the entire dataset, generating a machine learning score for each clip.

### Occupancy modelling

#### Comparison of different frameworks

We compared occupancy frameworks using data collected within Cockscomb and Manatee. For consistency between points, we filtered the dataset to retain only data from the first 28 days of sampling at each recorder point, and we additionally filtered the dataset to retain only data from the period coinciding with peak howling activity around dawn (4.00–7.00 am). We removed one sampling point that did not have 28 days of recordings leaving 58 points for the analysis.

We considered the collection of recordings from each point as an individual ‘site’. In line with other occupancy studies utilising ARU data processed by machine learning classifiers^15,24^, we defined true occupancy at each site as the sample-at-hand occupancy, determined by whether the species was present in at least one of all the recordings from that site. We defined* p* as the probability of detecting the species at a site given the species was present.

Sample-at-hand occupancy is typically estimated by manually verifying all the data used within the model^24^. Due to the high volume of data used in our models (over 200 h of recordings), it was not feasible to verify all the files used, so we employed a sampling approach to obtain our best estimate of sample-at-hand occupancy. We used a combination of sampling methods to minimise the chance of underestimating sample-at-hand. Sampling included: (1) review of the top-ten highest scoring files (global top ten) at each site, (2) review of the highest scoring file at each site on every third day, (3) review of files above a decision threshold of 0.77 (on the softmax scale) with early stopping when a presence was detected at each site, (4) scheduled listening to the first recording made every third day at each site, and (5) random listening of 2921 files (190 min). The threshold of 0.77 was chosen as it provided a good balance between recall and precision, with 85% recall and 92% precision on the validation data (Supplementary Fig. S1). This combination of sampling methods involving both random and targeted listening was used to ensure that, in addition to high-scoring files, files spanning a wide range of machine learning scores were verified at each site (Supplementary Fig. S2).

In total we reviewed 4,829 files (~ 5.5 h of recordings), with a mean of 58 files (SD = 27.7) reviewed per site and a maximum of 214 files reviewed at a single site. We considered the information gained from the extensive manual verification process as the best available estimate of ‘true’ parameter values, and that it represents the best estimate that could be achieved from the reduced dataset (i.e. reduced level of verification)^15^. To assess performance of each model we thus calculated the difference between model-estimated occupancy and the estimated sample-at-hand occupancy. While we acknowledge that this estimate is not perfect, it is the most accurate approximation of the ground truth achievable under the circumstances. Although we cannot completely rule out the possibility of underestimating the true occupancy, we took extensive steps to minimize this risk through diverse verification methods.

For initial comparisons of methods involving the use of thresholding (ii and iii) we used the decision threshold of 0.77. For continuous-score occupancy models we transformed classifier scores into the logit scale to satisfy model assumptions (Supplementary Fig. S1). We did not include environmental covariates for occupancy as we were primarily interested in comparing occupancy estimates, and furthermore their inclusion would have vastly increased the complexity of our model comparisons. We included environmental covariates to model detection probability, as we found that this was necessary to account for the unexplained variation in detection probability in our dataset. Data exploration confirmed that detection probability was associated with environmental covariates including forest cover, and that there was also a difference between the two survey grids (Supplementary Information). Furthermore, we found that the association with forest cover was different between the two survey grids. Consequently we modelled detection probability as a function of ‘forest’ (calculated as standardised mean value of forest height^44^ within a 2 km circular buffer around each point), ‘grid’ (categorical covariate with two levels), and an interaction term. The forest covariate ranged 8.2–23.6 m in Cockscomb (mean = 19.6 m, SD = 5.1), and 1.7–20.9 m in Manatee (mean = 14.1 m, SD = 5.2). We included this structure to account for heterogeneity in detection probability in all models going forward. For all false-positive models, we assumed that false-positive rate was constant across sites. All models were run in R version 4.2.0^45^.

##### Standard occupancy model

This approach involved manually verifying howler monkey presence from a small subset of files and using the resulting detection data in a standard single season occupancy model (referred to throughout as the ‘standard’ occupancy model). We compared several different approaches for selecting data to verify, including (1) verification of the file with the highest classifier score on every third day from each site, resulting in ten files verified per site (referred to from here on as ‘top-ten listening’), (2) verification of all files above a threshold of 0.77 on every third day from each site (referred to from here on as ‘thresholded listening’), (3) random verification of a single file on every third day from each site (resulting in ten files verified per site), and (4) scheduled listening (as described above). The same set of randomly verified files was used from here on whenever the use of random verification is referred to, and likewise whenever the use of top-ten files is referred to. These sampling approaches all resulted in verified files for ten separate days at each site. We subsequently constructed the detection histories by creating a separate survey replicate for each day and assigning a detection (1) to any site/day combination that had at least one file with a confirmed howler monkey detection.

Because howler monkey vocalisations are distinctive we assumed manual verification of files did not result in any false-positive detections^24^. Thus these data were suitable for the standard occupancy model^4^, where site specific occupancy, $z_{i}$ (occupied = 1, not occupied = 0), is assumed to arise from a Bernoulli distribution:

1 $$z_{i} \sim Bernoulli\left( \psi \right)$$

where $\psi$ is the probability of occurrence. As $z$ is not directly observed, we model the observation process, where the observed detection, $y$ at site $i$ during survey replicate $j$ also arises from a Bernoulli process conditional on the latent occurrence process if the species is present:

2 $$y_{i , j} \sim Bernoulli\left( {p_{i} , z_{i} } \right)$$

3 $$logit\left( {p_{i} } \right) = \alpha_{0} + \alpha_{1} \times forest_{i} + \alpha_{2} \times grid_{i} + \alpha_{3} \times forest_{i} \times grid_{i}$$

where if the species is present at site $i$, then $p_{i}$ is the probability of detecting the species. If the species is absent at site $i$, then $p_{i}$ equals 0 such that we assume there is no potential for a false-positive detection. We fit the occupancy models using the R package *Unmarked*^46^ and evaluated model fit using the MacKenzie-Bailey goodness of fit test^47^.

##### Binary false-positive occupancy model (Royle-Link model and extensions)

This approach involved extracting all files above a chosen decision threshold and declaring them as presences. As this process will result in false-positive detections, we used the Royle-Link false-positive occupancy model^17^, the simplest extension of the original occupancy model. In this model, we use the parameter $p_{11}$ to describe the probability of detecting a species given it is present ($y = 1 | z = 1$), and $p_{10}$ to describe the probability of a false-positive detection ($y = 1|z = 0$). The observation model is then altered to allow $y$ to arise from a Bernoulli distribution even when $z$ is 0:

4 $$y_{i , j} \sim Bernoulli\left( {p_{11, i} z_{i} + p_{10} \left( {1 - z_{i} } \right) } \right)$$

with detection probability given the species is present ($p_{11}$) modelled as a function of forest height and survey grid:

5 $$logit\left( { p_{11, i} } \right) = \alpha_{0} + \alpha_{1} \times forest_{i} + \alpha_{2} \times grid_{i} + \alpha_{3} \times forest_{i} \times grid_{i}$$

We constructed the detection history by creating a separate survey replicate for each day and assigning a detection (1) to any site/day combination that had at least one file with a classifier score above the decision threshold. This model has multimodal likelihood, resulting in identical support for different parameter values. We addressed this issue by constraining the parameters so that $p_{11} > p_{10}$, an assumption that is supported by the validation data at the chosen threshold^17^. We imposed this constraint by providing arbitrarily chosen starting values that align with this condition (0.7 for $p_{11}$ and 0.1 for $p_{10}$)^23^.

Extensions of this model accommodate additional sources of verified data^26^. We investigated two approaches for incorporating verified data, firstly the multiple detection method (‘multi-method’) design which involves treating verified data as ordinary occupancy data (i.e. only subject to false negatives), and modelling these data alongside the false-positive data in a joint occupancy model^26^. The second approach, the multiple detection state model (‘multi-state’) involves modelling the probability of three different detection states (no detection, uncertain detection, and certain detection), with an additional parameter $b$ which is the probability of an uncertain detection being classified as certain^26^. We implemented both models using the manually verified top-ten files from each site and the thresholded score data used for the Royle-Link model. For the multi-method model, we defined the method as a detection covariate as specified in Kéry and Royle (2020). We did not supply starting values for these models as the verified data alleviated multi-modality issues. We fit the models using the package *Unmarked*^46^.

##### False positive-occupancy model using detection counts

This model, first proposed by Chambert et al. (2018) and expanded on in Kéry and Royle (2020), uses the counts of detections derived from automated classification of acoustic data, $y_{i, j }^{a}$. This framework uses two independent data sources that are incorporated into separate observation models; binary false-positive data ($y_{i , j}$) and frequency data ($y_{i, j }^{a}$). In our case, detection data from the ARUs was used as the source for the frequency data, and as there was no independent data source available for the binary false-positive model, this component was removed from the code. The observation model for $y_{i, j }^{a}$ assumes that frequency of detections is a Poisson random variable, with a baseline rate of true positives $\lambda$, and a false positive rate $\omega$, where:

6 $$y_{i, j }^{a} \sim Poisson\left( {\lambda_{i} z_{i} + \omega } \right)$$

And $\lambda$ is modelled as a function of detection covariates, forest height and survey grid, as follows:

7 $$log\left( {\lambda_{i} } \right) = \alpha_{0} + \alpha_{1} \times forest_{i} + \alpha_{2} \times grid_{i} + \alpha_{3} \times forest_{i} \times grid_{i}$$

A further extension of this model allows for manual verification of a subset of the detection data to improve parameter estimates. However, model convergence with the addition of verification data was unstable, therefore, this version of the model was not implemented.

We created the detection history by summing the number of putative positive detections (i.e. all files above the chosen decision threshold) for each day. We ran the model in the R package *jagsUI* using Markov-chain Monte Carlo (MCMC) simulations^48^ (see Supplementary Information for details of model implementation). We ran the model using 3 chains with 33,000 samples, 3000 burn-in samples, and 5000 adaptation samples. We evaluated model convergence by inspecting trace plots and evaluating Gelman-Rubin statistics^49^, and assumed convergence when parameters had Gelman-Rubin statistics < 1.1.

##### Continuous-score occupancy models using classifier scores (Rhinehart et al. & Kéry and Royle)

These approaches use the classifier scores of each file, which are related to the likelihood that the target sound is present and bypasses the need to use a decision threshold. We compared two models using this approach which were independently developed by Kéry and Royle (2020) and Rhinehart et al. (2022). Both models incorporate a model of classifier scores into the occupancy framework, whereby scores are assumed to be derived from a normal distribution with a mean and standard deviation that vary by group $(g)$ (present or absent). If the file does not contain the species, then the score value $x_{k}$ for sample $k$ is assumed to be assigned by the classifier as:

8 $$x_{k} |g = 0 \sim Normal(\mu_{0} ,\sigma_{0} )$$

where $\mu_{0}$ and $\sigma_{0}$ are the mean and standard deviation of the scores returned by the classifier for files that do not contain the target species. If the file does contain the species, then the score is assumed to be assigned by the classifier as:

9 $$x_{k} |g = 1 \sim Normal\left( {\mu_{1} ,\sigma_{1} } \right)$$

where $\mu_{1}$ and $\sigma_{1}$ are the mean and standard deviation of the scores returned by the classifier for files that do contain the target species. The parameters of the score distribution reflect the classifier quality^24^. Classifiers that have a large difference between $\mu_{1}$ and $\mu_{0}$ ​ can better distinguish between the two groups. Similarly, classifiers with smaller values of $\sigma_{1}$ and $\sigma_{0}$ can achieve higher precision in distinguishing between the groups. Both models describe the overall distribution of scores as a two-component Gaussian mixture, comprising negative files and positive files. Where $z_{i } = 1$ (the site is occupied by the species), both components (positive files and negative files) are present in the mixture, with the positive component weighted by the call rate (and in the case of the Kéry model also by the false positive rate). In sites where $z_{i } = 0$ (the site is not occupied), only the component describing negative files is present. For both models a subset of verified files can be added to improve performance. For the Kéry model, the mixture model for scores is incorporated as an additional level in the detection-count false-positive model (model (iii) described above), whereas in the Rhinehart model, the model structure is more akin to the multiple detection methods model of Miller et al*.* (2011). For the Kéry model we used the same model adjustments detailed in (iii), including modelling the baseline detection rate of true positives ($\lambda$) as a function of the detection covariates. The Rhinehart model has an equivalent term, $\theta$, which describes the probability that a species appears in a file at an occupied site, which we modelled as follows:

10 $$logit\left( {\theta_{i} } \right) = \alpha_{0} + \alpha_{1} \times forest_{i} + \alpha_{2} \times grid_{i} + \alpha_{3} \times forest_{i} \times grid_{i}$$

We implemented the models using the scripts provided in each publication with some minor adjustments to accommodate our survey design and improve model convergence (Supplementary Information). The input data consisted of machine learning scores, sampled from the full dataset by selecting the first file from each ten-minute interval. This approach was used to speed up model running time and to aid model convergence issues that arose with the full dataset. As for the detection-count false-positive model, The Kéry continuous-score model failed to converge with the addition of verification data, therefore this version of the model was not implemented. We ran the Kéry model with 3 chains of 12,000 samples, with 2000 burn-in, 1000 adaptation, and a thinning rate of 2. We ran the Rhinehart models with and without verification data with 3 chains with 20,000 samples and 3000 burn-in. We checked convergence of all models using the Gelman-Rubin diagnostic.

#### Influence of decision thresholds on occupancy estimates

We investigated the influence of decision threshold on occupancy estimates by constructing detection histories using incremental decision thresholds between 0.01 and 0.99 with an interval of 0.03. We ran the binary false-positive models (Royle-Link, the multi-state, and multi-method) and the detection-count false-positive model (without verification data) using each dataset (see Supplementary Information for details of model implementation).

We additionally explored the influence of decision threshold on the occupancy estimate of the standard occupancy model. Our baseline threshold for this test was 0.77, as this was the minimum threshold above which we verified data. We selected thresholds between 0.77 and 1 using an interval of 0.001, and re-ran the standard occupancy model using only verified data above the chosen threshold.

#### Influence of verification method on occupancy estimates

The false-positive models included extensions to accommodate manually verified data, however it is not obvious what method to use to select verification data, and if the choice of method influences estimates. Previous studies have used annotations from randomly selected files^15,24^. For common and frequently vocalising species such as the howler monkey, there will be a relatively high probability of randomly selected files containing positive detections. However, for rare and infrequently vocalising species this process is unlikely to confirm many positive detections without substantially increasing the number of randomly selected files to verify. For such species, an alternative strategy focusing on verifying high-scoring files, which are more likely to contain true positives, may be beneficial.

We compared model estimates for the false-positive occupancy models with the addition of (i) 10 randomly verified files, and (ii) top-ten verified files. We did not include the detection-count or the Kéry continuous score false-positive models in this comparison as they failed to converge with verification data. We used a decision threshold of 0.01 for the binary false-positive models to allow the detection histories to accommodate the same set of randomly chosen files.

#### Influence of temporal subsampling regime on occupancy estimates

Temporal subsampling of the data may be required to meet the models’ assumption of independence of surveys, but it is not evident how to subsample ARU and classifier score data, or how the approach used influences estimates. We compared two subsampling approaches: (i) selecting the first file within a time interval (systematic first-file subsampling), and (ii) selecting the highest scoring file within a time interval (maximum score subsampling). We explored time intervals of 10 min and 30 min for both approaches. We compared estimates from the three binary false-positive models, the detection-count false-positive model, and the Rhinehart continuous-score false-positive models without verified data using these subsampling approaches. We omitted the Kéry continuous-score false-positive model from this comparison due to instability of model convergence. We used a decision threshold of 0.77 to construct the detection history for the binary and detection-count false-positive models. A summary of all model comparisons is found in Supplementary Table S1.

#### Efficiency of methods

We recorded computational running time for all methods. All computations were run on a Macbook M1 Pro Chip with 8-core CPU except for the CNN which was trained in Google Colab using one GPU.

## Results

### Classifier performance

The CNN effectively separated howler monkey vocalisations from background sounds (Supplementary Fig. S1). Classifications performed on the labelled validation data showed that distributions of positive and negative scores were well separated ($\mu_{0}$= − 2.2, $\sigma_{0} = 1.1$, $\mu_{1}$= 2.6, $\sigma_{1} = 2.3$).

### Estimated sample-at-hand occupancy

Manual verification of the 4,249 files sampled from all the data used in the models confirmed occupancy for 32 of the 58 sites (0.55). Naive occupancy from the annotated datasets included in the models was 5 sites (0.09) for the randomly annotated dataset (580 total files annotated), 11 sites (0.19) for the scheduled annotations (580 total files annotated), 21 sites (0.36) for thresholded listening down to the threshold of 0.77 every third day (759 total files annotated), and 21 sites (0.36) for annotation of the single highest scoring file every third day (580 total files annotated).

### Comparison of modelled occupancy estimates

**Fig. 2:**
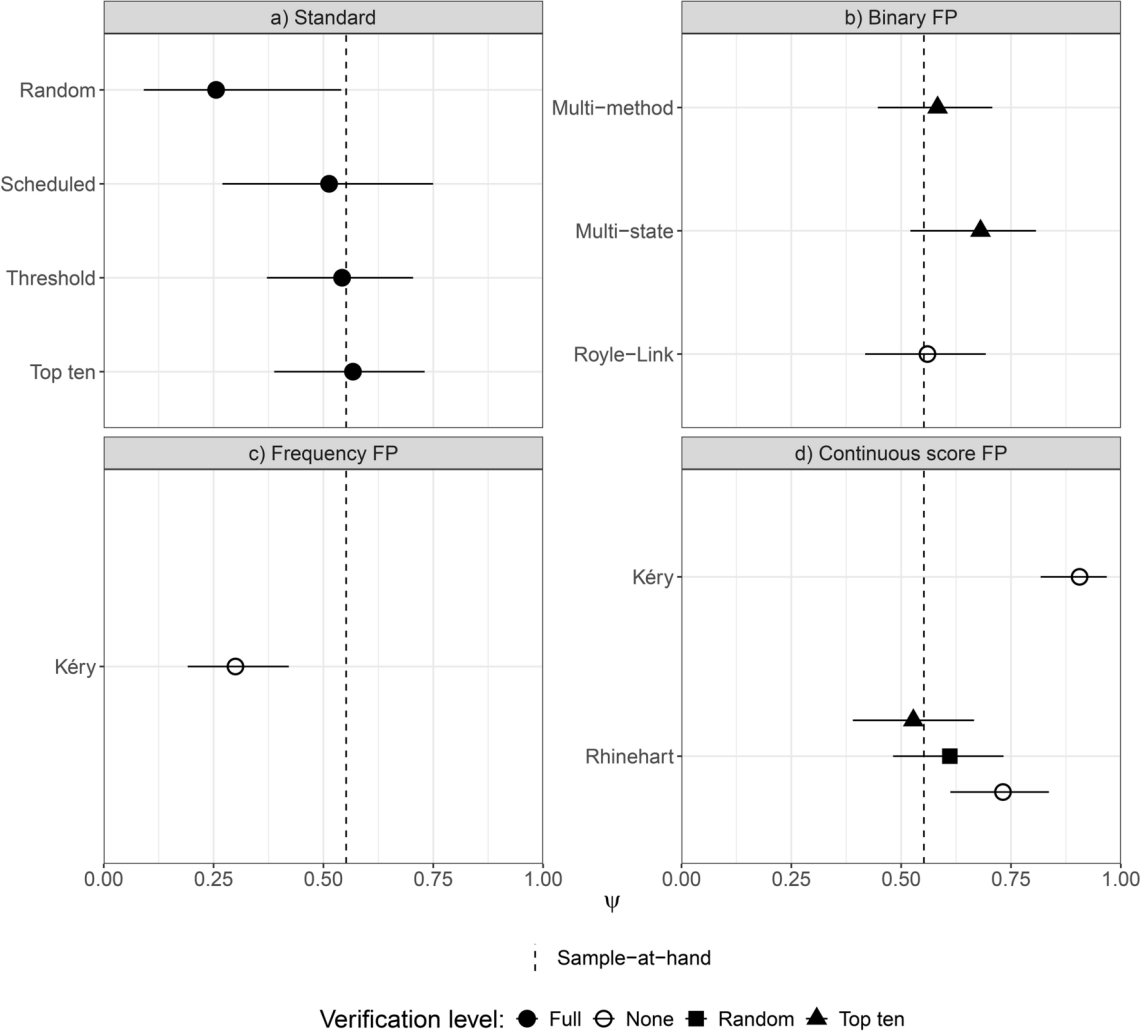
Comparison of modelled occupancy estimates produced by: (**a**) standard occupancy model, (**b**) binary false-positive model and extensions, (**c**) frequency false positive occupancy model using counts of detections, and (**d**) false positive occupancy models using raw classifier scores. Where applicable models were run with the addition of two different types of verified data from each site: 10 randomly selected files, and 10 files with the highest classifier score. A threshold of 0.77 was used for binary and frequency false-positive models. Results were removed for variations that did not converge (e.g. Kéry model with randomly verified data, and without verified data). Bars denote 95% confidence intervals and credible intervals for maximum likelihood and Bayesian approaches respectively. The dashed vertical line represents the sample-at-hand occupancy estimate.

The standard occupancy models using the top-ten data and thresholded listening data both produced occupancy estimates close to the sample-at-hand (Fig. 2). The thresholded model produced an estimate within 0.01 of the sample-at-hand ($\psi$ = 0.54, 95% confidence interval (CI) 0.37–0.70), and the top-ten model estimated occupancy at 0.02 over sample-at-hand ($\psi$ = 0.57, 95% CI 0.39–0.73). The standard occupancy model with scheduled listening produced an estimate 0.04 below sample-at-hand and with larger confidence intervals than the other standard occupancy models ($\psi$ = 0.51, 95% CI 0.27–0.75). The standard occupancy model with random data produced an estimate 0.30 below sample-at-hand ($\psi$ = 0.26, 95% CI 0.09–0.54).

Of the binary false-positive models, both the Royle-Link model and the multi-method model with top-ten verification data produced estimates similar to the sample-at-hand (Royle-Link $\psi$ = 0.56, 95% CI 0.42–0.69; multi-method $\psi$ = 0.58, 95% CI 0.45–0.71). The multi-state false-positive model with top-ten verification data produced an occupancy estimate 0.13 above sample-at-hand ($\psi$ = 0.68, 95% CI 0.52–0.81) (Fig. 2). The detection-count false-positive model underestimated occupancy by 0.25 in the absence of verification data ($\psi$ = 0.30, 95% credible interval (CRI) 0.19–0.42), and failed to converge with the addition of verification data.

The Rhinehart continuous-score false-positive model without the addition of verification data produced an estimate 0.18 above sample-at-hand ($\psi$ = 0.72, 95% CRI 0.60–0.83) (Fig. 2). With top-ten verification data, this model produced an estimate within 0.02 of sample-at-hand ($\psi$ = 0.53, 95% CRI 0.39–0.66). With the addition of randomly verified data, this model produced an estimate within 0.06 of sample-at-hand ($\psi$ = 0.61, 95% CRI 0.48–0.73). The Kéry continuous-score false-positive model produced an estimate 0.35 above sample-at-hand in the absence of verification data ($\psi$ = 0.91, 95% CRI 0.82–0.97). As for the detection-count false-positive model, this model failed to converge with the addition of verification data.

### Influence of decision threshold

**Fig. 3:**
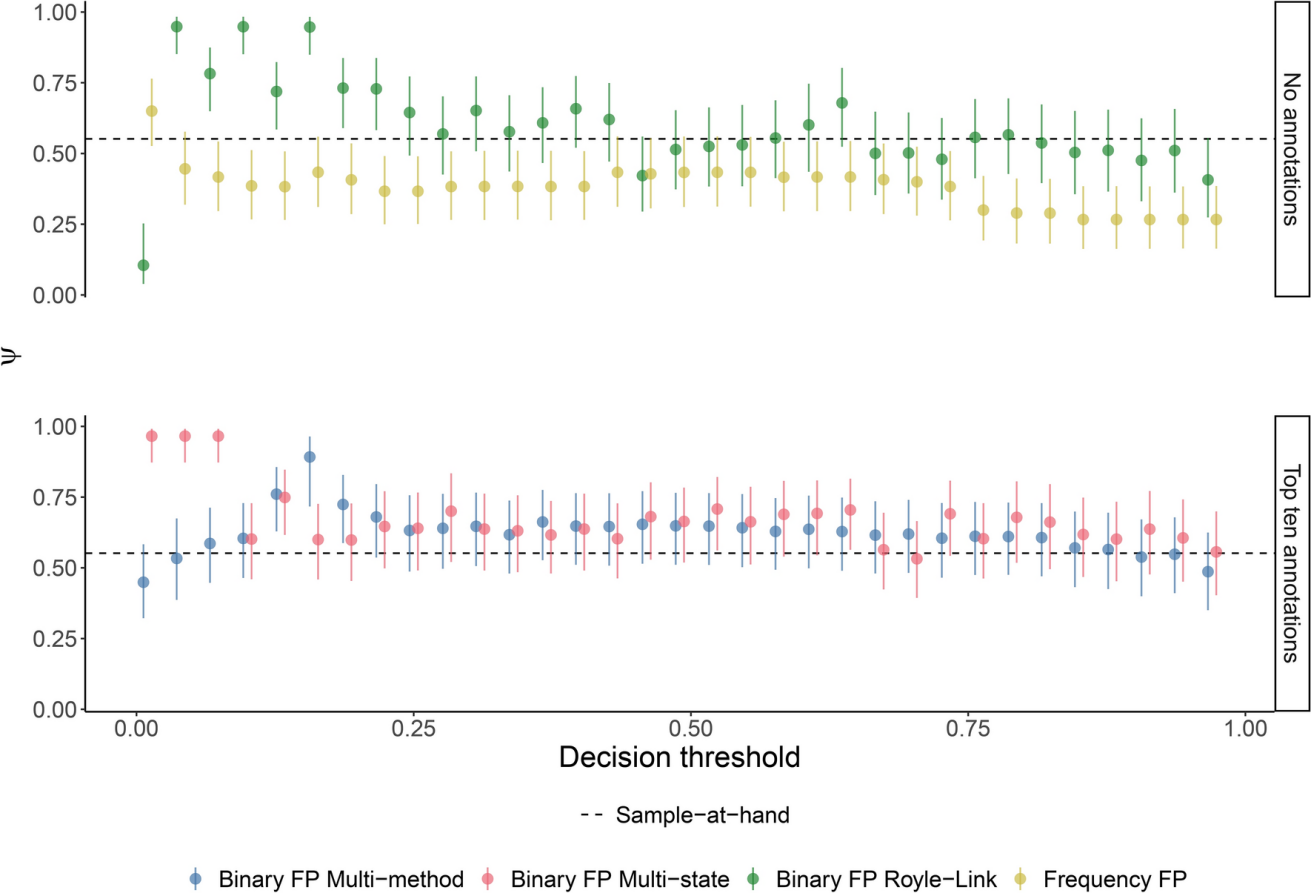
Influence of decision threshold on occupancy estimate using the detection frequency occupancy model (Chambert var.), the multi-method model, the multi-state model, and the Royle-Link false positive occupancy model. Models were run with and without the addition of verified data where applicable, which consisted of the top-ten files with the highest classifier score from each site. Bars denote 95% confidence intervals and credible intervals for maximum likelihood and Bayesian approaches respectively.

Estimates from the Royle-Link model remained relatively stable while the decision threshold was above 0.25, with estimates deviating by no more than 0.14 from the sample-at-hand. However, when the threshold dropped below 0.25, error increased, and the model tended to overestimate occupancy, with deviations reaching up to 0.45 from the sample-at-hand (Fig. 3). A similar pattern was observed for occupancy estimates derived from the multi-method and multi-state occupancy models, which had relatively stable estimates above a threshold of 0.25 (estimates within 0.11 and 0.16 of sample-at-hand for each model respectively), with increasingly unstable estimates below this threshold (estimates within 0.34 and 0.41 of sample-at-hand). Occupancy estimates produced by the detection-count false-positive model were generally more stable, however occupancy was consistently underestimated with the exception of the lowest threshold, 0.01, which produced an estimate 0.10 above sample-at-hand (Fig. 3).

The decision threshold had minimal impact on the standard occupancy model with thresholded data. Most positive detections had a classifier score of 0.95 or higher (mean = 0.98, SD = 0.04), so detection histories remained stable across thresholds from 0.77 to 0.95. Occupancy estimates within this range showed minimal variation, deviating by less than 0.01 from sample-at-hand occupancy. For thresholds above 0.95, positive detections in the detection histories became sparse as more zeros were introduced. The most notable changes to the detection histories occurred at thresholds exceeding 0.99. Occupancy estimates between 0.95 and 0.99 remained close to sample-at-hand, varying by less than 0.02. However, for thresholds beyond 0.99, the model’s occupancy estimates fluctuated, with deviations reaching up to 0.10 from sample-at-hand occupancy (Supplementary Fig. S3).

### Influence of verification method

**Fig. 4:**
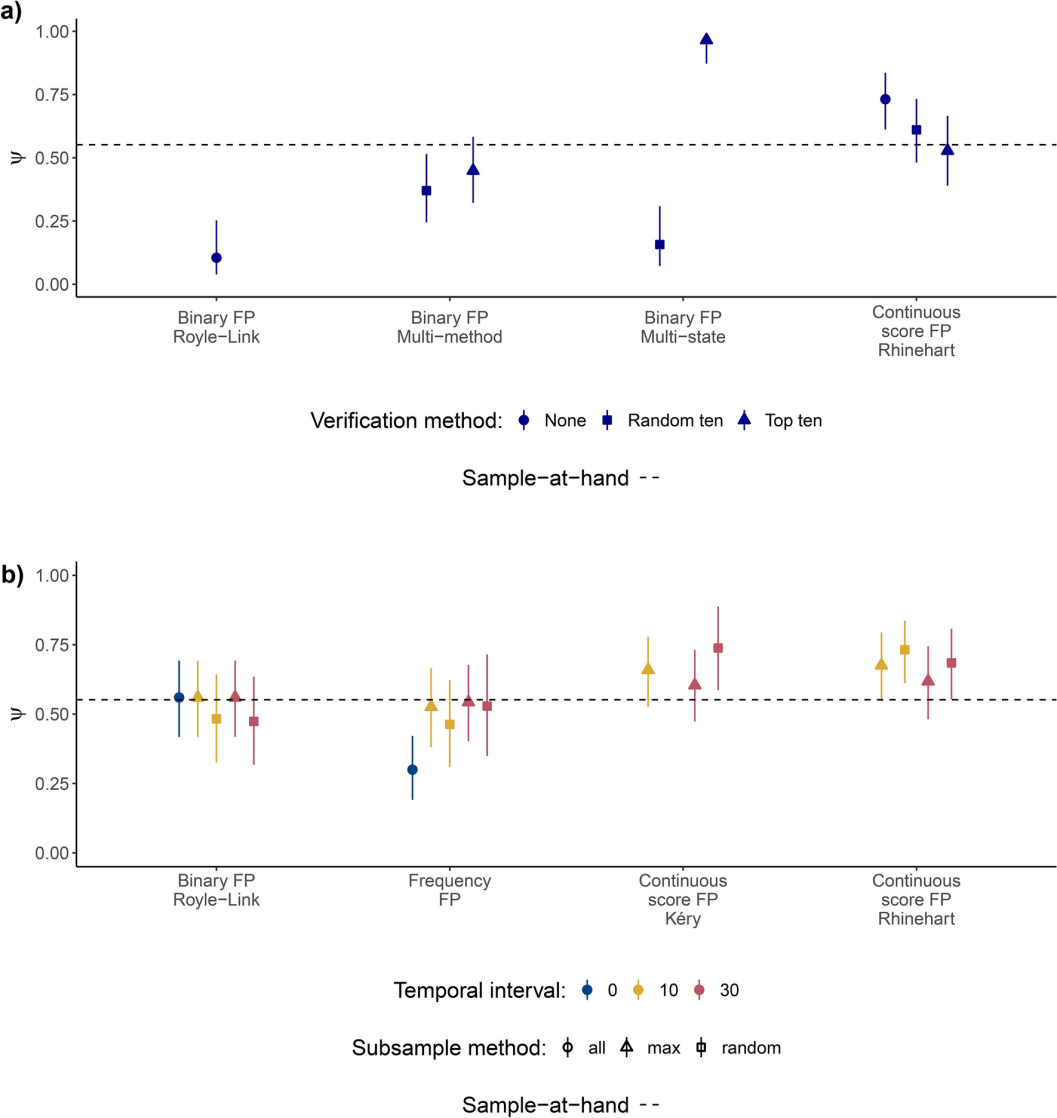
(**a**) Influence of data verification method on occupancy estimate. Verification methods include: no verification, 10 randomly selected files, and top-ten files with the highest classifier score. (**b**) Influence of temporal subsampling regime on occupancy estimate. Intervals used for subsampling were 0 (no subsampling), 10 min, and 30 min. Subsampling methods were either ‘random’, which involved selecting the first recording from each interval, or ‘max’ which involved selecting the file with the highest classifier score within each interval. Results were removed for variations that did not converge (e.g. several of the Kéry models). Decision thresholds of 0.01 and 0.77 were used for (**a**) and (**b**) respectively. Bars denote 95% confidence intervals and credible intervals for maximum likelihood and Bayesian approaches respectively.

The type of verification data used had the strongest effect on occupancy estimates for the multi-state occupancy model, which produced an estimate 0.41 above sample-at-hand with top-ten verification data ($\psi$ = 0.97, 95% CI 0.87–0.99) and produced an estimate 0.39 below sample-at-hand with randomly validated data e ($\psi$ = 0.16, 95% CI 0.07–0.31) (Fig. 4). In comparison, estimates from each of the remaining models were relatively similar to each other (for each model) regardless of verification method, but generally had lower error with top-ten verification data as opposed to randomly validated data.

### Influence of temporal subsampling regime

The temporal interval used for the subsampling regime had minimal impact on error of occupancy estimates compared to the influence of subsampling regime (e.g. maximum score subsampling versus systematic first-file sampling). For all models, error was lower with the use of maximum-score subsampling as opposed to systematic first-file sampling (Fig. 4). The Rhinehart continuous-score model did not converge without temporal subsampling, so this model run was excluded from the results.

### Efficiency of approaches

Training the CNN took 115 min and predictions on 5400 h of data took 7.4 h using parallel processing on 4 cores and using a single GPU. Manual verification of 10 files per site took 15 min. The most computationally demanding models were Bayesian false-positive models, in particular the Kéry continuous-score false-positive model which took 35 min to run. Running time for the remaining Bayesian models ranged up to 12 min, and all other models ran in under one minute (Supplementary Table S2). The detection-count and Kéry continuous-score false-positive models encountered significant convergence issues, likely due to the absence of marginalization in the model code^50^. As a result, their convergence was highly unstable, often requiring multiple re-runs with varying initial values to improve convergence. Despite these efforts, both models failed to consistently converge when verification data were included.

## Discussion

Evaluating and comparing modeling approaches is essential for developing practical recommendations for integrating ARU data and machine learning outputs into occupancy models. In this study, we addressed this need using the Yucatán black howler monkey as a case study. Our findings demonstrated that classifier-guided listening, combined with a standard occupancy model, offers an effective and practical method for accurately estimating occupancy. This approach was particularly efficient when paired with the top-ten verification strategy, which further reduced verification effort. False-positive models also produced accurate estimates but only under specific conditions, such as particular combinations of decision thresholds and temporal subsampling regimes. The Royle-Link binary false-positive model produced accurate estimates within a realistically conservative range of decision thresholds, as did the multi-method and the Rhinehart continuous-score false positive models when combined with top-ten verification data, indicating that these methods can be effective in specific scenarios. Nevertheless, the dependence of false-positive models on factors like decision thresholds and temporal subsampling regimes, for which the most suitable approach cannot be determined a priori, limits the practical application of these methods.

Our results demonstrated the effectiveness of classifier-guided listening, which focuses listening effort on files with high classifier scores, for estimating site occupancy. We employed two approaches to classifier-guided listening: one involved listening to all files above a pre-set threshold (thresholded listening) for each replicate survey, and the other involved listening to a reduced dataset consisting of the top-scoring file from each replicate, leading to the review of just ten 4-s files per site (top-ten approach). Both approaches produced similarly accurate occupancy estimates within a standard occupancy model, with the top-ten approach minimising the required listening time. In the absence of classifier-guided listening, occupancy estimates were less accurate and precise. Random verification led to substantial underestimation, while scheduled listening produced an occupancy estimate slightly below the sample-at-hand, with wider confidence intervals. The particular effectiveness of classifier-guided listening combined with a standard occupancy model in our study was likely due to the combination of a high-performance classifier and a readily detectable study species. In scenarios with low classifier performance or reduced target species detectability, this approach may become less efficient, requiring significantly more listening time to confirm detections. In such cases, there is potential that false-positive models may offer more suitable alternatives.

The false-positive models produced occupancy estimates comparable in accuracy to the standard occupancy models, however their performance was influenced by factors such as decision threshold, temporal subsampling, and verification strategies. In general, most of the false-positive models achieved higher accuracy with the use of a maximum-score temporal subsampling approach, whereby the file with the highest machine-learning score was subsampled, the use of a mid-range decision threshold (where applicable), and supplemented with top-ten verification data (where applicable). These findings, however, are likely specific to this case study.

Sensitivity to decision thresholds poses challenges for both standard and false-positive occupancy models^20^. As demonstrated by Knight et al*.* (2019) for standard occupancy models, high decision thresholds reduce detection probability, which can lead to biased occupancy estimates. Furthermore, heterogeneity in detection probability, caused by variation in score distributions across sites, may be exacerbated at higher thresholds and further contribute to bias. In our study, however, estimates from the standard occupancy model with thresholded listening data remained relatively robust to changes in decision threshold, with minimal error observed across all thresholds. This resilience is likely due to the high classifier performance and detection rates in our system, which may mitigate the impact of threshold variation within a standard occupancy model.

Our results demonstrated that false-positive models are also sensitive to the choice of decision threshold, which can significantly affect the accuracy and stability of occupancy estimates. In theory, these models should remain unaffected by decision thresholds if their assumptions hold true across all thresholds. However, in practice, these assumptions are often not fully met, leading to variations in model performance. This was evident in our results, where estimates from the binary false-positive models fluctuated with changes to decision threshold, with the highest error rates occurring at lower thresholds where the false-positive rate was greatest. In contrast, the frequency false-positive model provided relatively stable estimates across thresholds but consistently underestimated occupancy. For most false-positive models in our study, using a mid-range threshold of 0.50 or above generally yielded stable estimates. However, it is unclear whether this threshold would be appropriate in scenarios with differing classifier performance or species detectability. Users of these models may need to evaluate estimates across a range of thresholds to ensure stability before applying these methods. A suitable range of thresholds can be determined by validating the classifier’s performance on a test dataset and assessing precision and recall across decision thresholds. Identifying a balanced range, where neither recall nor precision is significantly compromised, may provide a practical starting point for selecting suitable thresholds for these models.

An additional factor influencing estimator error for false-positive models was the incorporation of verification data. While verification data are often essential for model convergence^51^, there is limited guidance on how to sample or select the subset of data to verify. We compared two strategies: random verification and targeted verification using top-ten data. For the Rhinehart continuous-score and binary multi-method false-positive models, both verification strategies improved occupancy estimates, but targeted verification produced more accurate results. In contrast, the binary multi-state model substantially underestimated occupancy with random verification and overestimated occupancy with targeted verification data. Notably, this comparison was conducted at a very low decision threshold, which contributed to instability in occupancy estimates. At higher thresholds, the multi-state model produced estimates comparable in accuracy to the multi-method model when incorporating top-ten verification data, although tended to overestimate occupancy. In general, the impact of the verification strategy used likely depends on how the data are incorporated into the models. In frameworks where verification data inform the occupancy state process (e.g. the multi-method model), maximizing detections of true positives through targeted listening is beneficial. However, where verification data inform the detection process (e.g. the multi-state model), targeted verification may bias false-positive rate estimates, and potentially inflate occupancy estimates. Future research should explore alternative strategies, such as stratified random sampling across machine learning score bands, to provide a more balanced representation of false positives and true positives^52^.

Our case study highlighted several challenges in fitting false-positive models to field data. The basic Royle-Link false-positive model and its extensions are difficult to fit due to multimodal likelihoods^17^, which can result in label switching between parameters^53^. Supplying unambiguous data or restricting starting values is often essential for these models to converge^51^; consequently, many of the models necessitated exploring multiple combinations of initial values. While convergence of the Royle-Link model was straightforward with restricted starting values, we encountered significant difficulties with more complex Bayesian models, particularly the detection-count and continuous-score false-positive models from Kéry and Royle (2020). Convergence for these models was especially unstable when verified data were included, likely due to the absence of marginalization, as discussed by Augustine et al. (2023). These challenges, combined with high computational demands and sensitivity to subjective choices such as initial values, limit the practical utility of these models. In our case, these more complex approaches offered no clear advantage over a standard occupancy model with a small subset of verified data, making their implementation both costly and unnecessary.

Our case study, though not fully generalizable, highlights a growing trend in bioacoustics: leveraging machine learning models to process large datasets, often collected for other purposes, to estimate species occupancy. For a high-performing classifier and a readily detectable species, classifier-guided listening combined with a standard occupancy model provided accurate occupancy estimates while minimizing verification effort. This efficient approach can also supply verification data for certain false-positive occupancy models. While false-positive occupancy models also produced accurate estimates under specific conditions, their performance was highly sensitive to subjective choices, such as decision thresholds, temporal subsampling, and verification strategies. The challenge of predefining stable parameters, coupled with increased computational complexity, reduces the practicality of these models. Further studies are needed to evaluate these factors across diverse systems and to develop general guidelines for parameter selection. Through this case study, we provide initial evidence on model performance, laying the groundwork for establishing generalizable recommendations for integrating machine learning outputs with occupancy models.

## Supplementary Information

Supplementary Information.

## Data Availability

Data and model code are provided on figshare at: https://doi.org/10.6084/m9.figshare.23309159 and https://doi.org/10.6084/m9.figshare.23308730.
