# Supplementary material for: A comparison of statistical methods for deriving occupancy estimates from machine learning outputs
Source: Sci Rep. 2025 Apr 27;15:14700. doi: 10.1038/s41598-025-95207-3 (PMC12034756; doi:10.1038/s41598-025-95207-3)
Supplement: Supplementary file 1 — Supplementary Information. [file 41598_2025_95207_MOESM1_ESM.pdf]

Supplementary Information

Supplementary Information for:

Katsis, LK; Rhinehart, TA; Dorgay, E.; Sanchez, E.; Snaddon, JL; Doncaster, CP; Kitzes, J. A comparison of statistical methods for deriving occupancy estimates from machine learning outputs

Data and model code are provided on figshare at: https://doi.org/10.6084/m9.figshare.23309159 and https://doi.org/10.6084/m9.figshare.23308730

Methods

Machine learning classifier

To assemble a training dataset of howler monkey clips and background sounds, we took a random sample of 1000 four second files collected in Tapir Mountain Nature Reserve (TMNR), and 1000 four second files sampled from Manatee, and manually labelled the clips as present or absent. From this sample, we obtained 71 positive clips (58 from TMNR, and 13 from Manatee), and 1929 absent clips (3.55 % overall positive). We randomly split this dataset into training and validation sets (70% for training, and the remaining 30% for validation of the classifier). We supplemented the training dataset with howler monkey recordings obtained from the Macaulay Library at the Cornell Lab of Ornithology (see acknowledgements for file accreditations). We used Raven Lite software (Raven Lite, 2022) to manually annotate howler monkey sounds from seven files of varying length, and subsequently created an additional 40 four-second positive files which we included in the training data. As canonical machine learning algorithms assume equal distributions between each class (Krawczyk, 2016), we balanced our training dataset to a ratio of 1:1 (present: absent) by oversampling the presence files to match the number of background sounds in the training data. We validated the model on unbalanced data to assess its performance on a realistic dataset.

We trained a convolutional neural network (CNN) classifier in Python using OpenSoundscape version 0.7.1 (Lapp et al., 2023) in a Google Colab notebook using one GPU. Our model consisted of the ResNet18 CNN architecture. Our preprocessing pipeline involved creating spectrogram images from the audio clips, applying augmentations, and converting the image to a PyTorch Tensor for model training. We created spectrograms using a window length of 512 and overlap of 256. Spectrograms were bandpassed to include only the frequency bands between 0 - 3500 Hz. Augmentations included in the pipeline comprised: i) image overlay, which blends randomly selected negative class images to each positive sample in the training dataset; iii) time mask, iv) frequency mask, v) addition of Gaussian noise to the tensor; and vi) random affine transformation to the image. These augmentations were predominantly required to alter recordings from the Macaulay dataset to make them more closely resemble noisy field data. We used the default model training parameters provided by OpenSoundscape. We trained the model for 50 epochs (iterations over the dataset) and selected the epoch with the highest MAP score (mean precision score) on the validation data.

We evaluated model performance by performing predictions on the validation dataset. The model produced scores (-inf, +inf) for presence and absence of each sound file. We evaluated model performance by comparing the distribution of these scores to the true class labels within the validation dataset.

Occupancy models

Heterogeneity in detection probability

Exploration of the data using standard occupancy models with no covariates for detection probability or occupancy indicated unmodeled heterogeneity in detection probability and violating a key assumption of occupancy models. This was evidenced by poor model fit using the MacKenzie-Bailey goodness of fit test and high c-hat values for models using all annotated datasets. For example, the fit statistic for the standard occupancy model with no covariates for the annotated top-ten dataset had a p value of 0.001, and an estimated c-hat of 16.34. Detection heterogeneity can result from several factors, including different availability for detection at each site, differing trigger rates of the ARUs at each site, differing abundance at each site, in addition to fine-scale habitat covariates (Kays et al., 2021; Royle, 2006). We investigated the inclusion of detection covariates that accounted for different triggered recording rates at each site, a random effect for each site, and a fixed effect of the classifier score of the file (or mean score if multiple files) during each survey replicate, however none of these approaches was effective for modelling detection heterogeneity, as evidenced by failure to validate using the MacKenzie-Bailey goodness of fit tests.

Differing abundance is frequently a cause of detection heterogeneity, so we explored environmental covariates at each site that may be related to abundance of howler monkeys. We found that the frequency of detections was related to the survey grid, and forest cover, and within each survey grid there was a different relationship between forest cover and detection rates. This interaction occurred because one of the survey grids which was characterised by low elevation, and a high gradient of forest cover, had a strong positive association between forest cover and howler monkey detection rate, whereas the other survey grid, which was characterised by high elevational gradient and high forest cover, had a negative relationship with forest cover. This negative relationship was due to the fact that howler monkeys are not found at high elevations, and elevation is positively correlated with forest cover. We subsequently confirmed that including an interaction effect between survey grid and forest cover effectively accounted for detection heterogeneity within our models.

Implementation of Bayesian models

The information below details the priors, initial values, and MCMC settings that we used to implement the Bayesian models detailed in the manuscript. We chose these settings using an iterative process that began with using the same priors and model specification to reflect those in the literature from where we sourced the models (Kéry and Royle (2020) for the detection-count false-positive model and the Kéry continuous-score false-positive model) and (Rhinehart et al., 2022) for the Rhinehart continuous-score false-positive models. We then visually inspected trace plots for a sample of these models and adapted the specification where necessary to achieve model convergence.

i) False positive-occupancy model using detection counts

We used the model code as provided in Kéry and Royle (2020), with some minor changes to accommodate our dataset. Firstly, as the original model framework has two observation models for two separate data sources, regular false-positive data in addition to detection counts, and we only had one source of data (detection counts), we adjusted the code to remove the model for the binary data. Additionally, we adjusted the baseline rate of true positives, λ, to be a function of environmental covariates as follows:

$$log(\lambda_i) = \alpha_0 + \alpha_1 \times forest_i + \alpha_2 \times grid_i + \alpha_3 \times forest_i \times grid_i \quad (1)$$

We used uninformative priors of Uniform(0,1) for ψ, Uniform(0,1000) for ω, and N(0,100) for the α coefficients. We used initial values of 1 for z, and selected initial values of Uniform(0,0.4) for ω. The remaining values were initialized automatically by JAGS. We ran the model using 3 chains with 5000 adaptation, 33,000 iterations, a thinning rate of 2, and 3000 burn-in. We assessed convergence using r-hat values and visual inspection of trace plots.

ii) Kéry and Royle (2020) Continuous-score occupancy model

We adjusted the model code provided in Kéry and Royle (2020) using the same adjustments as specified for the detection-count model, namely removal of the binary false-positive model, and modelling λ as a function of environmental covariates. We made further adjustments to allow σ to vary between the distributions of μ_0 and μ_1 . We additionally adjusted the prior distributions of μ_0 and μ_1 to N(0, 10^{12}) and N(1, 10^{12}), and we adjusted the prior distributions of the standard

deviations of both score distributions to Uniform(0,1000). These alterations were necessary to improve convergence and to more accurately reflect the true score distributions. We used the same priors as specified for the detection-count model.

We used initial values of 1 for z, and selected initial values of Uniform(1,2) for ω. We used initial values of μ_0, μ_1, σ_0, and σ_1 derived from the classifier's score distribution on the validation data ($\mu_0 = -2.6$, $\mu_1 = 2.3$, $\sigma_0 = 1.1$, and $\sigma_1 = 2.3$). We additionally provided initial values for group membership, g, whereby the file contained the target species ($g = 1$) if the score was above 0, and did not contain the target species ($\mathrm{g} = 0$) if the score was below 0. The remaining values were initialized automatically by JAGS. We ran these models using 3 chains with 1000 adaptation, 12,000 iterations, thinning rate of 2, and 2000 burn-in. We assessed convergence using r-hat values and visual inspection of trace plots.

iii) Rhinehart et al. continuous-score occupancy model

We used the model code as provided in (Rhinehart et al., 2022), with some minor adjustments. Firstly, the model code was adjusted to accommodate a random number of files per site. Secondly, the model was adjusted to accommodate the addition of the detection covariates for θ which is the equivalent of detection probability/baseline true positive rate in this model, as follows:

$$logit(\theta_i) = \alpha_0 + \alpha_1 \times forest_i + \alpha_2 \times grid_i + \alpha_3 \times forest_i \times grid_i$$

We used uninformative priors of Uniform(0,1) for ψ, and Uniform(0,1) for the mean value of θ, which is the probability of a file containing the target species. The prior for the intercept, α_0, was calculated as the logit of the mean value for θ. Priors for α_1, α_2, and α_3 were drawn from Normal(0,2). As with the previous models, we initially used uninformative priors for the covariates, drawn from N(0,1000) however we could not achieve model convergence with these priors. We used priors of N(0,10^4) for the mean of the distribution of scores for files where the species was absent, N(1,10^4) for the mean of the distribution of scores for files where the species was present, and Uniform(0,10^4) for the standard deviations of both score distributions.

We selected initial values of Uniform(0,1) for ψ, and and we used initial values of μ_0, μ_1, σ^1, and σ^2 derived from the classifier's score distribution on the validation data. We selected initial values for α_1, α_2, and α_3 from N(1,2), and α_0 as the logit of Uniform(0,1). We used initial values

of 1 for z where the true z value was not known from annotations; in which case it was provided as z data within the model. We ran these models using 3 chains with 30,000 iterations and 3000 burn-in and assessed convergence using r-hat values and visual inspection of trace plots.

Literature Cited

Kays, R., Hody, A., Jachowski, D.S., Parsons, A.W., 2021. Empirical evaluation of the spatial scale and detection process of camera trap surveys. Mov. Ecol. 9, 41. https://doi.org/10.1186/s40462-021-00277-3

Kéry, M., Royle, J.A., 2020. Applied hierarchical modeling in ecology: analysis of distribution, abundance and species richness in R and BUGS: Volume 2: dynamic and advanced models. Academic Press.

Krawczyk, B., 2016. Learning from imbalanced data: open challenges and future directions. Prog. Artif. Intell. 5, 221–232. https://doi.org/10.1007/s13748-016-0094-0

Lapp, S., Rhinehart, T., Freeland-Haynes, L., Khilnani, J., Syunkova, A., Kitzes, J., 2023. OpenSoundscape: an open-source bioacoustics analysis package for Python. Methods Ecol. Evol. n/a. https://doi.org/10.1111/2041-210X.14196

Raven Lite, 2022. Raven Lite: Interactive Sound Analysis Software (Version 2.0.4).

Rhinehart, T.A., Turek, D., Kitzes, J., 2022. A continuous-score occupancy model that incorporates uncertain machine learning output from autonomous biodiversity surveys. Methods Ecol. Evol. 13, 1778–1789. https://doi.org/10.1111/2041-210X.13905

Royle, J.A., 2006. Site occupancy models with heterogeneous detection probabilities. Biometrics 62, 97–102.

Figures

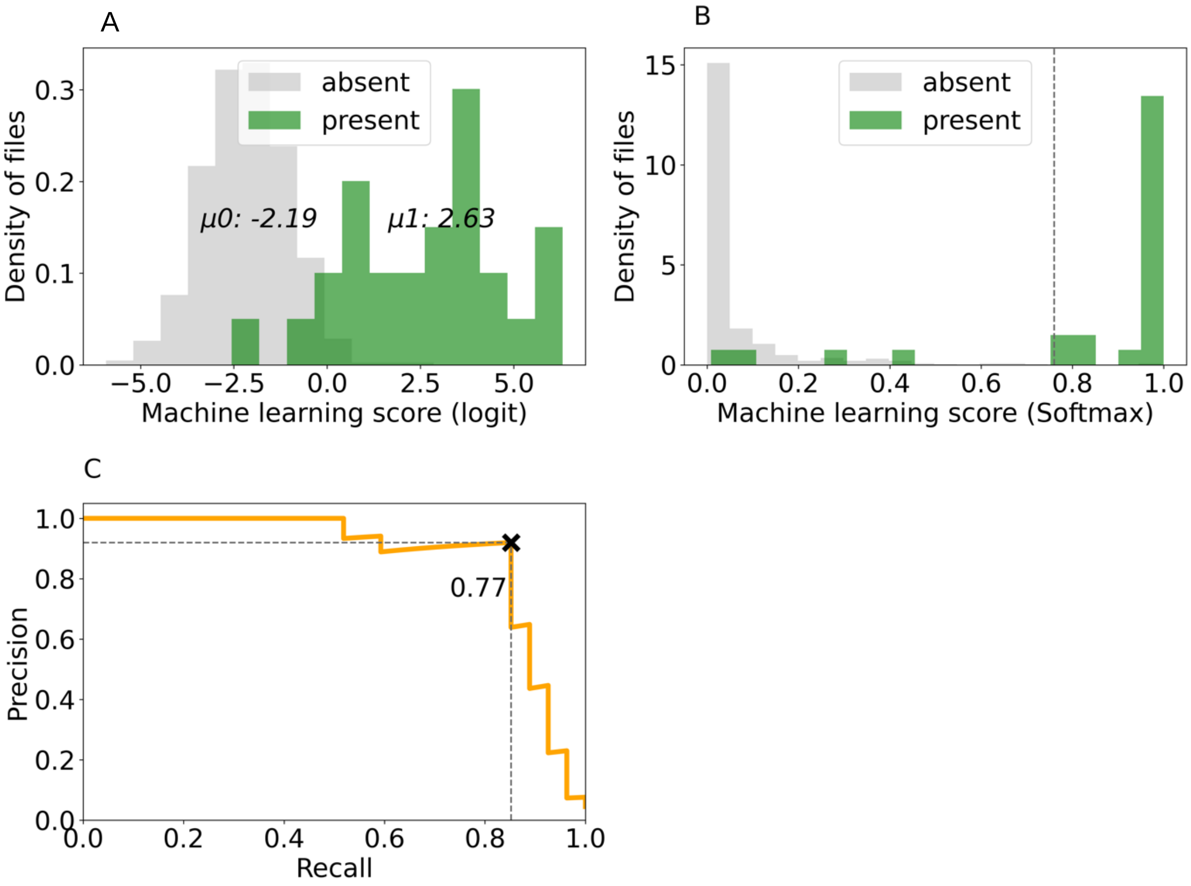

Figure S1

(A) Distribution of classifier scores on the logit scale for howler monkey presence, with true labels of the validation data represented by the colours of the bars. **(B)** Distribution of classifier scores on the softmax scale for howler monkey presence, with true labels of the validation data represented by the colours of the bars. **(C)** Precision recall curve showing varying precision and recall of howler monkeys associated with different decision thresholds on the softmax scale. Black crosses denote the decision thresholds referred to in Figure 3. The threshold of 0.77 was identified by choosing the threshold with precision of over 90%.

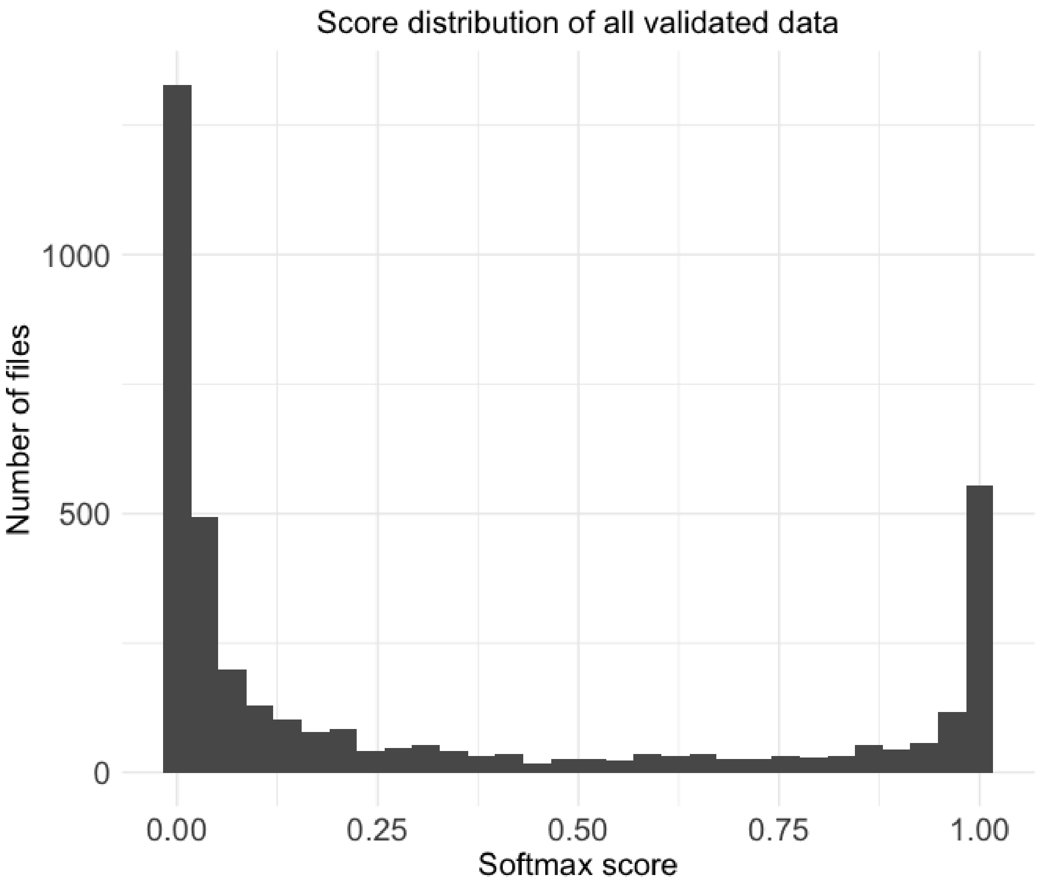

Figure S2

Distribution of machine learning scores for the 4,829 validated files.

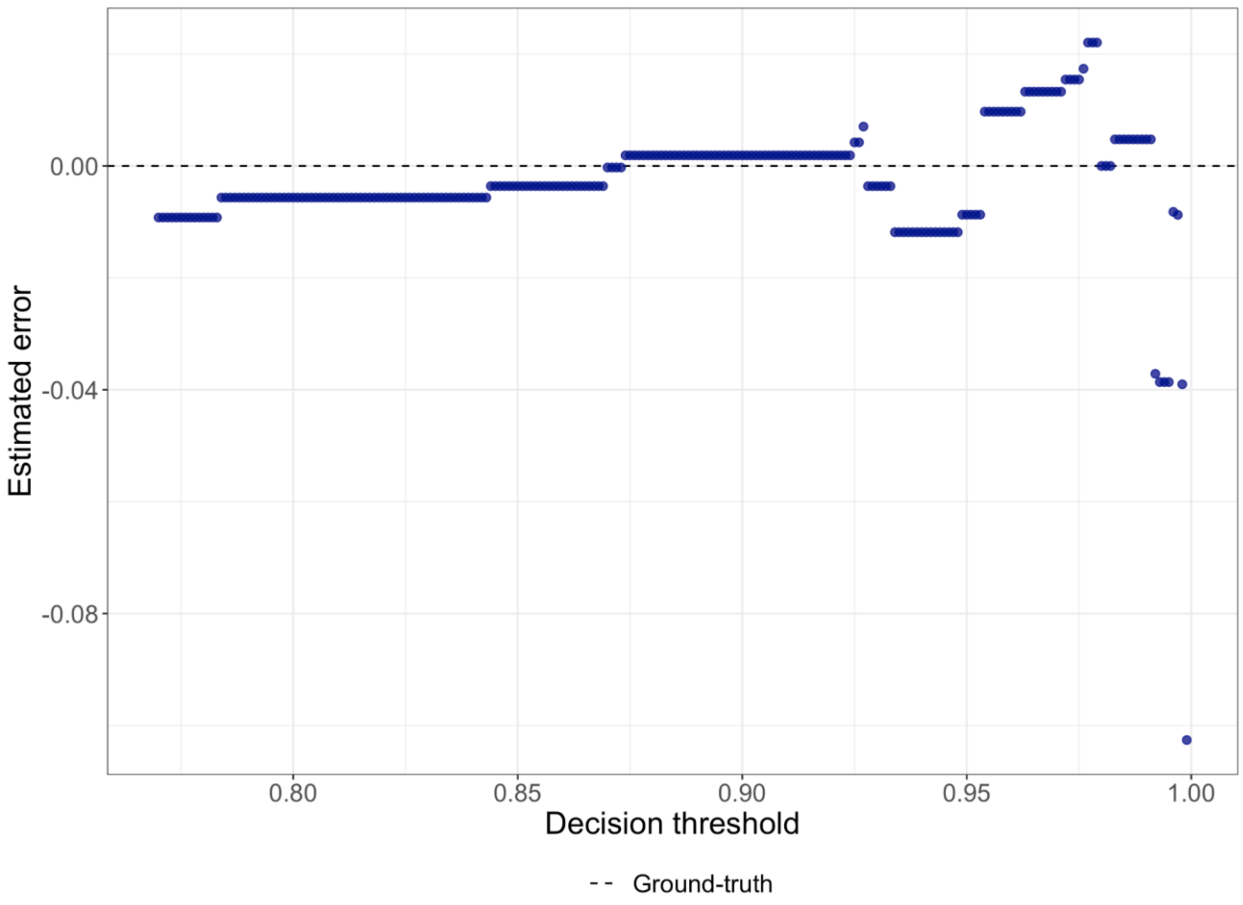

Figure S3

Influence of decision threshold on occupancy estimate using the standard occupancy model with ‘thresholded listening data’, which was compiled by manually verifying all files above a minimum threshold on every third day at each point.

Tables

Table S1
Summary of the model comparisons conducted.

**Model name (as used in paper)**	**Input data**	**Comparisons**			**Model source**
		**Decision threshold**	**Temporal sample**	**Verification strategy**	
Standard occupancy model	Presence-absence data (false negatives only)	✓		✓	Mackenzie *et al.* (2002)
Royle-Link model	Presence-absence data with false positives	✓	✓		Royle and Link (2006)
Multi-method model	Presence-absence data with false positives and verification data	✓	✓	✓	Miller *et al.* (2011)
Multi-state model	Presence-absence data with false positives and verification data	✓	✓	✓	Miller *et al.* (2011)
Detection-count false-positive model	Counts of detections with false positives (no verification data)	✓	✓		Kéry and Royle (2020)
Rhinehart continuous-score false-positive model	Raw classifier score data for every clip (with and without target species) with and without verification data		✓	✓	Rhinehart *et al.* (2022)
Kéry continuous-score false-positive model	Raw classifier score data for every clip (with and without target species, no verification data)				Kéry and Royle (2020)

Table S2

Computation time for each model (ran on a Macbook M1 Pro Chip with 8-core CPU). Data from 28 days monitoring at 58 sites, equating to over 200 hours of audio. Estimates are provided using a decision threshold of 0.77 for the binary and detection-count false-positive occupancy models.

Model	Verification level	Psi (lower, upper)	Est. error	Computation time (mins)
a) Standard				
Standard w/top ten	Full	0.57 (0.39, 0.73)	0.02	0.00
Standard w/threshold	Full	0.54 (0.37, 0.70)	-0.01	0.00
Standard w/scheduled	Full	0.73 (0.01, 1.00)	0.18	0.00
Standard w/random	Full	0.26 (0.09, 0.54)	-0.30	0.00
b) Binary FP				
Royle-Link	None	0.56 (0.42, 0.69)	0.01	0.02
Multi-method w/top ten	Top ten	0.58 (0.45, 0.71)	0.03	0.00
Multi-state w/top ten	Top ten	0.68 (0.52, 0.81)	0.13	0.00
c) Detection-count FP				
Detection-count	None	0.30 (0.19, 0.42)	-0.25	0.72
d) Continuous-score FP				
Kéry	None	0.91 (0.82, 0.97)	0.35	35.30
Rhinehart	None	0.73 (0.61, 0.84)	0.18	11.40
Rhinehart	Random ten	0.61 (0.48, 0.73)	0.06	7.41
Rhinehart	Top ten	0.53 (0.39, 0.67)	-0.02	6.99
